# Optimization of κ-Selenocarrageenase Production by *Pseudoalteromonas sp*. Xi13 and Its Immobilization

**DOI:** 10.3390/molecules27227716

**Published:** 2022-11-09

**Authors:** Yashan Deng, Xixi Wang, Hui Xu, Cui Liu, Ran Li, Yuanyuan Zhang, Changfeng Qu, Jinlai Miao

**Affiliations:** 1 Department of Pharmaceutical Engineering, College of Chemical Engineering, Qingdao University of Science and Technology, Qingdao 266042, China; 2 Key Laboratory of Marine Eco-Environmental Science and Technology, First Institute of Oceanography, Ministry of Natural Resources, Qingdao 266061, China; 3 State Key Laboratory Base for Eco-Chemical Engineering in College of Chemical Engineering, Qingdao University of Science and Technology, Qingdao 266042, China; 4 Laboratory for Marine Drugs and Bioproducts of Qingdao National Laboratory for Marine Science and Technology, Qingdao 266071, China; 5 Marine Natural Products R&D Laboratory, Qingdao Key Laboratory, Qingdao 266061, China

**Keywords:** κ-selenocarrageenase, selenium oligosaccharides, lyophilized enzyme, immobilized enzyme, *Pseudoalteromonas sp.* Xi13

## Abstract

The bioenzymatic production of selenium oligosaccharides addresses the problems resulting from high molecular weight and poor water solubility of κ-selenocarrageenan, and lays foundation for its application as adjuvant drugs for cancer treatment and food additive. κ-selenocarrageenase extracted from *Pseudoalteromonas sp*. Xi13 can degrade κ-selenocarrageenan to selenium oligosaccharides. The maximum optimized κ-selenocarrageenase activity using Response Surface Methodology (RSM) was increased by 1.4 times, reaching 8.416 U/mL. To expand applications of the κ-selenocarrageenase in industry, the preparation conditions of it in either lyophilized or immobilized form were investigated. The activity recovery rate of the lyophilized enzyme was >70%, while that of the immobilized enzyme was 62.83%. However, the immobilized κ-selenocarrageenase exhibits good stability after being reused four times, with 58.28% of residual activity. The selenium content of κ-selenocarrageenan oligosaccharides degraded by the immobilized κ-selenocarrageenase was 47.06 µg/g, 8.3% higher than that degraded by the lyophilized enzyme. The results indicate that the immobilized κ-selenocarrageenase is suitable for industrial applications and has commercial potential.

## 1. Introduction

As a trace element, selenium is a component of glutathione peroxidase, which protects cell membranes from oxidative damage [1]. Compared with inorganic selenium, organic selenium can improve tissue selenium content and has high biological activity [2]. Recently, κ-selenocarrageenan extracted from the cell walls of various marine organisms have been reported to possess many biological and physiological activities such as anticoagulant [3], antithrombotic [4], anti-inflammatory [5], antiviral [6], and antitumor activities [7] shown to result from immunoactivation [8]. However, as an organic macromolecule of selenium polysaccharide, κ-selenocarrageenan has poor water solubility and therefore failed to be used [9]. Compared with κ-selenocarrageenan polysaccharides, oligosaccharides have higher selenium content and better bioavailability, which greatly overcomes the defects of κ-carrageenan [10]. Not only that, microbes are used to prepare κ-selenocarrageenan oligosaccharides in mild reaction conditions with advantages of easy control, environmental friendliness and high efficiency [11]. Carrageenan can be degraded into oligosaccharides by carrageenase from marine bacteria, including *Vibrio* [9], *Cytophaga* [12], *Alteromonas* [13], *Pseudomonas* [10]. However, few strains have been reported to produce κ-selenocarrageenase [11].

The improvement of living standards and environmental pollution in the 21st century have made people pay more attention to food safety and hygiene. Enzyme preparation used in the process of catalytic processing is a kind of bioactive substance which can be processed into different purities and dosage forms, including immobilized enzymes and cells obtained by immobilization technology [14]. With advantages of less energy consumption and economic cost, vacuum freeze-drying has been widely used in food in recent years to expand the commercial application of enzymes [15]. In addition, the enzyme is usually fixed in the carrier by physical and chemical methods to obtain immobilization; for example, the protease is bound to the carrier by covalent bond and non-covalent bond [16]. The immobilized enzyme can overcome the disadvantages of poor stability and low utilization of free enzyme, and can be reused many times [14].

In a previous report, we have isolated a *Pseudoalteromonas sp.* Xi13 strain producing κ-selenocarrageenase from the floating ice in Antarctica. In addition, TLC (Thin layer chromatography) analysis showed that the degradation products were mainly disaccharides and tetrasaccharides which displayed antitumor activity against Hela cells by determining the cell viability using Sulforhodamine B (SRB) method [17]. However, κ-selenocarrageenase preparations are rarely reported and their industrial application is problematic. In the present research, the fermentation conditions for producing κ-selenocarrageenase from *Pseudoalteromonas sp.* Xi13 were optimized by using response surface methodology (RSM). In addition, lyophilized and immobilized κ-selenocarrageenases were obtained and their storage stabilities were investigated. Moreover, the selenium content of products degraded by two enzyme preparations was compared to obtain the optimal preparation. The bioenzymatic production of selenium oligosaccharides has the advantages of mild reaction conditions, easy control, and high efficiency. It addresses the problems of high molecular weight and poor water solubility of κ-selenocarrageenan, and lays a foundation for its application as adjuvant for cancer treatment and food additive.

## 2. Results and Discussion

### 2.1. κ-Selenocarrageenase Activity and Response Surface Test Results

**Figure 1:**
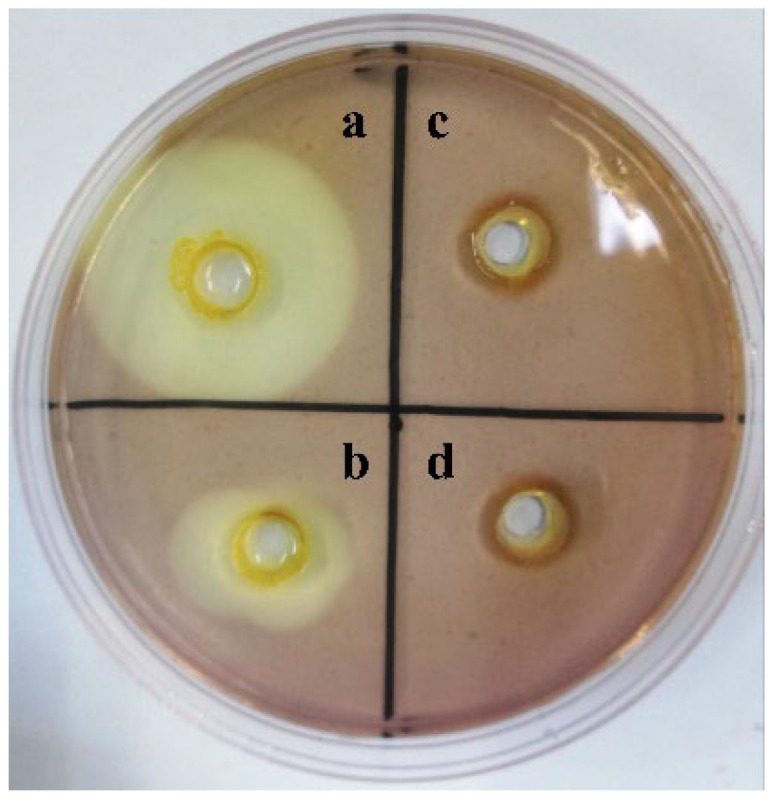
Screening results of κ-carrageenan degradation effect. (**a**): intracellular enzyme; (**b**) extracellular enzyme; (**c**): bovine serum protein; (**d**): PBS buffer.

Polysaccharides can only be stained with Lugol’s iodine solution (a mixture of potassium iodide and iodine components), while oligosaccharides degraded by carrageenases cannot be stained. As shown in Figure 1, the transparent zones around intracellular and extracellular enzymes appeared on the plate, thus proving that κ-selenocarrageenan was degraded by κ-selenocarrageenases. The intracellular and extracellular enzyme activities of Xi13 were 6.079 ± 0.5 U/mL and 3.254 ± 0.3 U/mL, respectively.

Design Expert 8.0.6 software was used to conduct quadratic multiple regression analysis of variance on the experimental results. The quadratic regression equation is the following:

*Y* = 8.33 + 0.43*A* − 0.39*B* − 0.38*C* − 0.46*AB* − 0.46*AC* − 0.18*BC* − 1.95*A*^2^ − 1.25*B*^2^ − 0.44*C*^2^. (1)

The model’s *R*^2^ value was 0.9730 with a 95% confidence level, indicating that the quadratic polynomial model (Equation (1)) fits the experimental data well. Furthermore, the lack of fit (*p* = 0.1552 > 0.05) was non-significant, indicating that the model was acceptable and suitable for the optimization of enzyme production in the fermentation of Xi13. The positive linear effects of *A* (κ-selenocarrageenan concentration), *B* (Ca^2+^ concentration), and *C* (temperature) on *Y* (enzyme activity) were found to be significant (*p* < 0.05). All of the quadratics were significant items (*p* < 0.05), yet those of *BC* were non-significant (*p* > 0.05) (Table 1). The model showed good fitting with the actual results, and may be used to analyze and predict the enzyme activity of Xi13.

**Figure 2:**
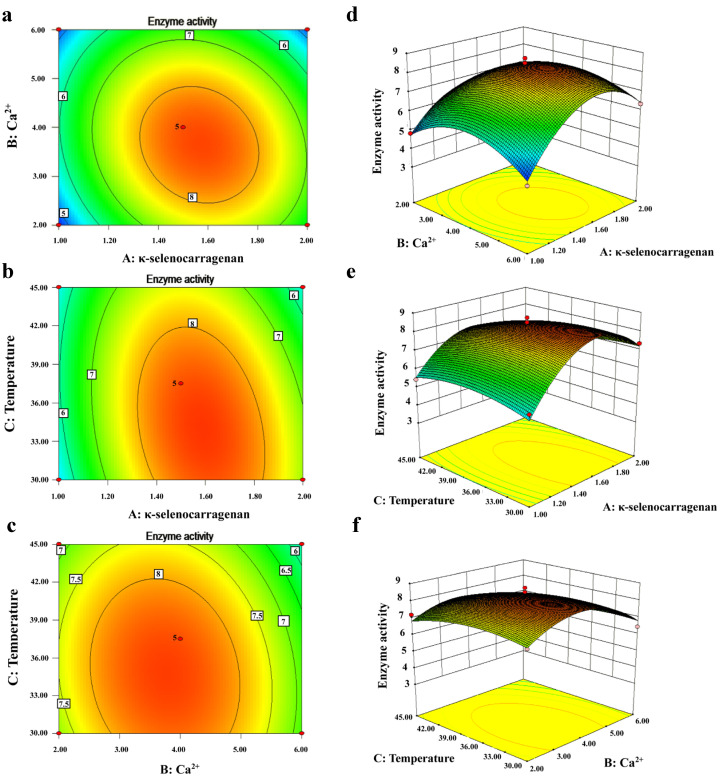
Response surface (**a**–**c**) and contour plots (**d**–**f**) of the effects of κ-selenocarrageenan concentration (*A*), Ca^2+^concentration (*B*) and temperature (*C*) on κ-selenocarrageenase activity.

The Expert Design software was used for further analysis and prediction, and the response surface map and contour map were drawn as Figure 2. κ-selenocarrageenase activity increased with the three factors, with its peak activity approaching the midpoint of the response plot. As shown in Figure 2, the optimum concentration of κ-selenocarrageenan for enzyme production was 1.59%. The results were similar to the marine strain on *Pedobacter sp. NJ-02* [9]. When the concentration is <1.59%, the enzyme activity increases with the increase in the concentration, because the effect of the increase in the concentration on microbial growth, metabolism and inductive effect of κ-selenocarrageenase is greater than the adverse effect due to the increase in the viscosity of the reaction medium. When the concentration is higher than this value, however, the enzyme activity decreases with the increase in the concentration, because the adverse effect due to the increase in the viscosity on microorganisms is greater than that of the increase in the concentration. Generally, when fermentation encounters high viscosity media containing macromolecules, the large mass transfer resistance will seriously affect the growth and metabolism of microorganisms [18]. A high concentration of κ-carrageenan will result in a high viscosity of the medium because the viscosity significantly increases with the increases in the concentration. A high viscosity will lead to a large mass transfer resistance of the substrate, such as oxygen and κ-carrageenan, thereby reducing the enzyme production rate of strain Xi13. Ca^2+^ concentration was found to have significant effects on κ-selenocarrageenase production and cell growth. The optimum concentration of Ca^2+^ is 3.69 mmol/L, which was consistent with a previous report by Guo et al. [19] showing the optimum concentration of Ca^2+^ for the production of κ-carrageenase by *Thalassospira sp.* Fjfst-332 was 0.1 g/L.

To sum up, the three factors corresponding to the highest enzymatic activity of the response surface map were 1.6% κ-selenocarrageenan (theoretical value 1.59%), 3.7 mmol/L Ca^2+^ (theoretical value 3.69 mmol/L), and the temperature 33 °C (theoretical value 32.52 °C), respectively. The maximum predictive activity was 8.495 U/mL, increased by about 1.4 times compared with the original enzyme activity (6.079 U/mL). In comparison with 2.14 U/mL of optimized κ-carrageenase production by *Cellulosimicrobium cellulans* [20] investigated, the optimized enzyme activity of Xi13 with 8.495 U/mL was significantly higher. Three parallel fermentation experiments were repeated, and the enzyme activities were 8.372, 8.407 and 8.468 U/mL, respectively. The average value was 8.416 U/mL, which is in good agreement with the predicted value (8.495 U/mL), indicating that the model could predict the κ-selenocarrageenase production (Table 2) very well.

### 2.2. Enzymatic Properties of κ-Selenocarrageenase

**Figure 3:**
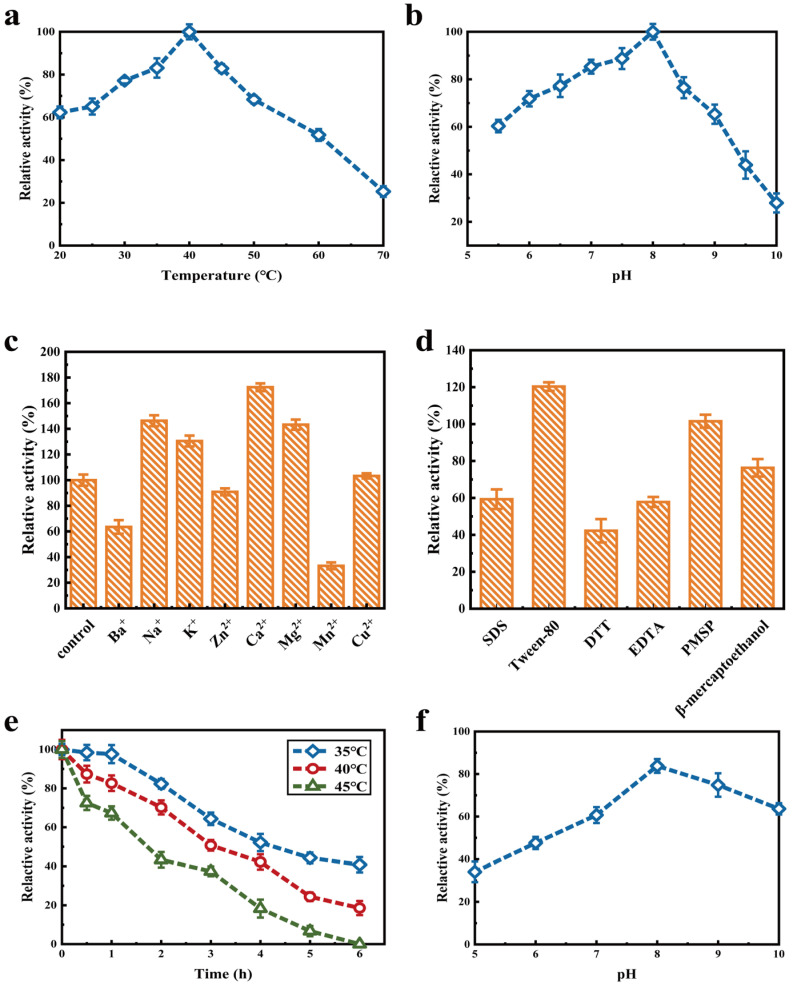
Enzymatic properties of κ-selenocarrageenase: effects of temperature (**a**), pH (**b**), metal ions (**c**), and chemical additives (**d**); its thermal stability (**e**) and pH stability (**f**).

As shown in Figure 3a, with the increase in temperature, the catalytic capacity of κ-selenocarrageenase increased first and then decreased, and the activity of κ-selenocarrageenase reached the highest value at 40 °C. The relative enzyme activity at 70 °C was more than 70% lower than that at the optimum temperature (Figure 3a). Figure 3b shows that the enzyme activity was completely lost at 45 °C for 6 h. While under 35 °C and 40 °C for 6 h, the relative enzyme activities were still remained 40.78% and 18.56%, respectively. The optimum temperature for thermostable κ-carrageenase production of *Pseudoalteromonas sp.* QY203 was 45 °C [21]. Sun et al. [22] isolated a strain *Tamlana sp.* HC4 producing carrageenase whose optimum reaction temperature was measured to be 30 °C. Compared with above researches, the thermal stability of κ-selenocarrageenase from Xi13 needs to be further improved.

The pH is an important factor that affects the enzyme activity. As shown in Figure 3c, the relative activity of κ-selenocarrageenase was maximized at pH 8.0, which was ~1.7-fold that at pH 5.5, indicating that the enzyme had better tolerance to alkaline environment. This is probably because the spatial structure of the protein changed under acidic conditions, leading to the loss of its function. However, the relative enzyme activity value at pH 10 was only about 30% of that at the optimal pH 8.0. It is suggested that the strong alkali may cause the change of spatial conformation of the protein, thus leading to the inactivation of the protein, resulting in inhibited rate of enzymatic reaction. It also could be observed that the relative enzyme activity remained >70% when kept at 4 °C, pH 8.0–9.0 for 3.0 h, indicating the optimum pH of κ-selenocarrageenase from Xi13 was 8.0 (Figure 3d). The results were similar to previous findings on κ-carrageenases of *Pseudoalteromonas sp.* ZDY3 [23] and *Tamlana sp.* HC4 [22].

As shown in Figure 3e, the activity of κ-selenocarrageenase can be promoted by Na^+^, Ca^2+^, K^+^ and Mg^2+^. While Ca^2+^ has the best effect at the same concentration of each ion, which can increase the activity by 72.43%. Mn^2+^, Zn^2+^, and Ba^2+^ at 5.0 mmol/L concentration inhibited the enzyme activity to some degree, while Mn^2+^ has the strongest inhibitory effect with only 33.21% residual activity. Cu^2+^ had no obvious effect on enzyme activity. However, Mg^2+^ and Ba^2+^ significantly stimulated the κ-carrageenase activity of *Pseudoalteromonas porphyrae* [24], which is inhibited by Ca^2+^, Zn^2+^, Cu^2+^ and Na^+^. The reason may be that ions have different effects on the functional domains of proteins due to different structures of kinds of carrageenases.

It can be seen from Figure 3f that the surfactant Tween-80 promotes the activity of κ-selenocarrageenase because it can increase the contact between enzyme and substrate and interact with enzyme molecules through hydrogen bonding and hydrophobic interaction [25]. EDTA inhibits the enzyme activity, indicating that the enzyme was ion dependent [26]. DTT and β-mercaptoethanol has different inhibitory effects on the enzyme, which may be due to the presence of thiol groups and the protective effect of catalytic amino acid (Cys). SDS is a denaturing agent, which makes enzyme conformation change greatly and thus inhibits its activity. However, PMSF shows no significant effect on enzyme activity, indicating that serine and histidine are not involved in the active center [27].

### 2.3. Optimization of Vacuum Freeze-Drying Process

**Figure 4:**
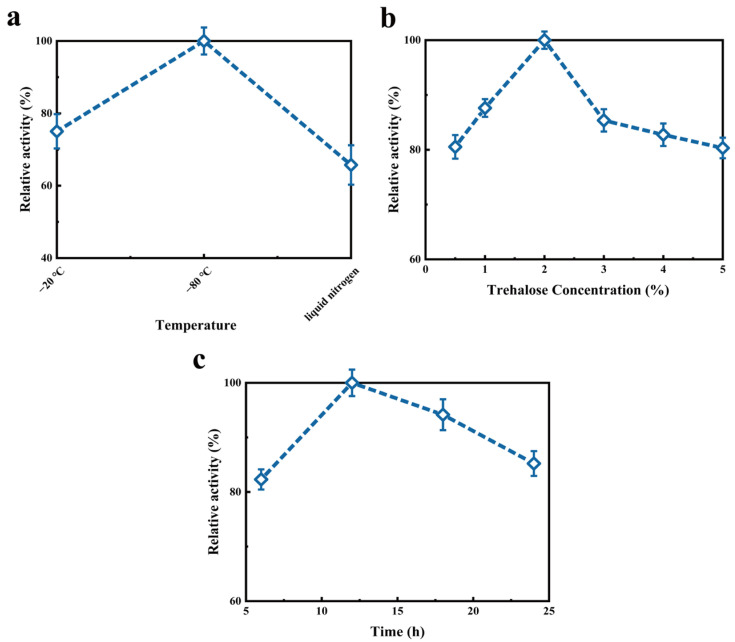
Effect of technological parameters including (**a**) pre-freeze temperature, (**b**) pre-freeze time and (**c**) trehalose concentration on κ-selenocarrageenase activity in freeze-drying process.

**Figure 5:**
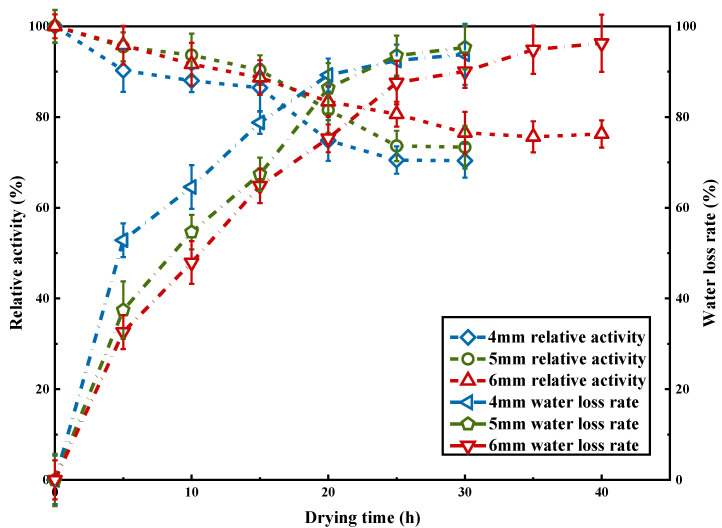
κ-selenocarrageenase activity and water rate of loss vs. drying time.

The relative enzyme activity of κ-selenocarrageenan pre-frozen solution at −20 °C, −80 °C and liquid nitrogen is shown in Figure 4a. It can be seen from Figure 4a that the optimum activity was reached at −80 °C, while the enzyme activity decreased when the temperature deviated from −80 °C. When the temperature reaches between the freezing point and the eutectic point of the product, the enzyme needs to cool quickly. When the temperature is too low, the pre-freezing rate will slow down, which will easily lead to protein denaturation [28]. The temperature of −20 °C is not enough to allow the enzyme to cool quickly while the pre-freezing rate slows down due to the low temperature with liquid nitrogen, so −80 °C is chosen as the pre-freezing temperature. As can be seen from Figure 4b, the relative enzyme activity increased gradually when the pre-freezing time was from 6 h to 12 h, and reached the optimum activity at 12 h. Prolonging the pre-freezing time would reduce the enzyme activity, thus 12 h should be chosen as the optimal pre-freezing time. In the process of vacuum freeze-drying, the enzyme activity increased with the increase in the thickness of enzyme solution layer when it was <5 mm (Table 3). However, the enzyme activity began to decrease when the thickness was >5 mm. As the thickness increases, the mass transfer resistance of water vapor will increase, which will slow the leakage of water vapor, thus reducing the damage caused by rapid dehydration to proteins [29]. Nevertheless, when the feed liquid layer is too thick, the heat transfer resistance and the mass transfer resistance of water will be increased, resulting in the extension of the drying stage and loss of enzyme activity [30]. Trehalose as a lyophilized protective agent can stabilize the structure and function of proteins. With the increase in trehalose concentration, the enzyme activity reached the highest at the concentration of 2.0% (Figure 4c). When the concentration exceeded 3%, the enzyme activity tended to be stable. Too much protective agent will increase the viscosity of the solution, prolong the freeze-drying time and waste energy, so the concentration of 2.0% trehalose was chosen. The changes in κ-selenocarrageenase activity and the water rate of loss with the increase in drying time are shown in Figure 5. κ-Selenocarrageenase is only frozen rather than dried initially. It can be seen that there is no loss of the enzyme activity during the freezing process, and the main stage of loss is in the process of water sublimation. As thermodynamic instability occurs during drying, the hydrogen bond is broken between protein and water to make enzyme activity decrease [28].

### 2.4. Stability of Lyophilized κ-Selenocarrageenase

**Figure 6:**
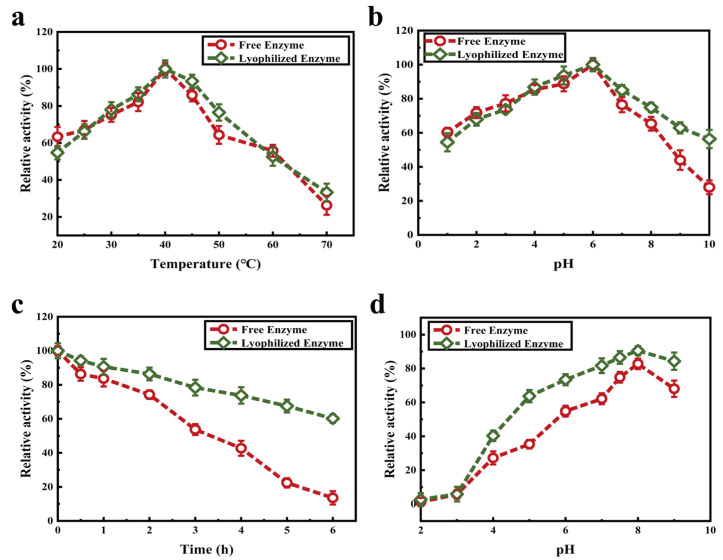
Enzymatic properties of lyophilized enzyme and free enzyme: effects of temperature (**a**) and pH (**b**); their thermal stability (**c**), and pH stability (**d**).

The optimum catalytic temperature of free enzyme is the same as that of the lyophilized enzyme, and the change trend of their activities is similar (Figure 6a). However, the relative enzyme activities of the free and lyophilized enzyme were 13.56% and 60.17%, respectively, after holding at 40 °C for 6.0 h (Figure 6b). The thermal stability of lyophilized enzyme was better than that of free enzyme at 40 °C due to the protective effect of lyophilized protectant on protein. The optimum pH of lyophilized enzyme was pH 8.0, which was the same as that of free enzyme (Figure 6c). However, the free enzyme was more sensitive to the change of pH than the lyophilized enzyme. When the pH changes slightly, the free enzyme activity decreases or increases rapidly. The relative enzyme activity of lyophilized enzyme remained >80% when kept at 4 °C and pH 7.0–9.0 for 3.0 h (Figure 6d). The results indicate that the pH stability of lyophilized enzyme is higher than that of free enzyme.

**Figure 7:**
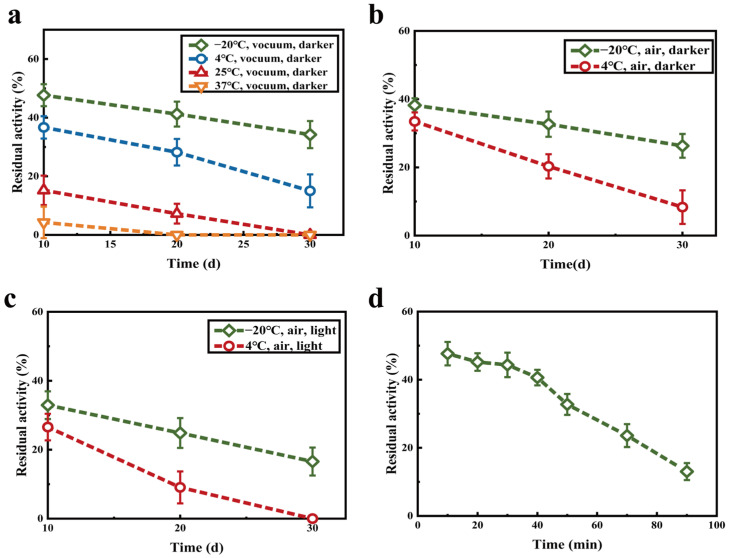
Storage stability test of lyophilized enzyme: effects of temperature (**a**), interaction of temperature and air (**b**), interaction of temperature, air and light (**c**), and UV irradiation with 100 μm/cm^2^ (**d**).

There are various factors such as temperature, pH, air and light that contribute to storage stability of enzyme. After 30 days of storage at −20 °C and 4 °C, the residual enzyme activity was 34.22% and 15.02%, respectively. By contrast, the enzyme activity was completely lost when kept at 25 °C and 37 °C for 30 days (Figure 7a). The spatial structure of the enzyme is likely to change resulting in loss of enzyme activity due to the high temperature during storage. Therefore, −20 °C is chosen as the storage temperature of lyophilized enzyme. Not only temperature but also air significantly affects the storage stability of enzyme. The residual enzyme activities of lyophilized enzyme were 26.31% and 13.34% at −20 °C and 4 °C when exposed to air and protected from light for 30 days (Figure 7b), respectively, which were about 10% lower than those under vacuum conditions. Therefore, vacuum storage should be selected because air has an adverse impact on the storage of enzyme preparations. Figure 7c shows that the residual enzyme activity of lyophilized enzyme stored at −20 °C in light for 30 days is 16.56%, which was lower than that under dark condition. Since light provides energy to accelerate enzyme inactivation, enzyme preparations should be stored away from light [31]. In order to control the microbial content, sterilization such as UV irradiation is necessary, which inevitably affects enzyme activity [32]. When the UV irradiation time was within 40 min, the activity of lyophilized enzyme remained in a stable range, and the residual enzyme activity was >40%. The activity began to decrease at 40 min of UV irradiation, and decreased to 13.03% of that at 90 min (Figure 7d), indicating that the activity could be maintained if the irradiation time was kept within 40 min.

### 2.5. Optimization of Enzyme Immobilization

**Figure 8:**
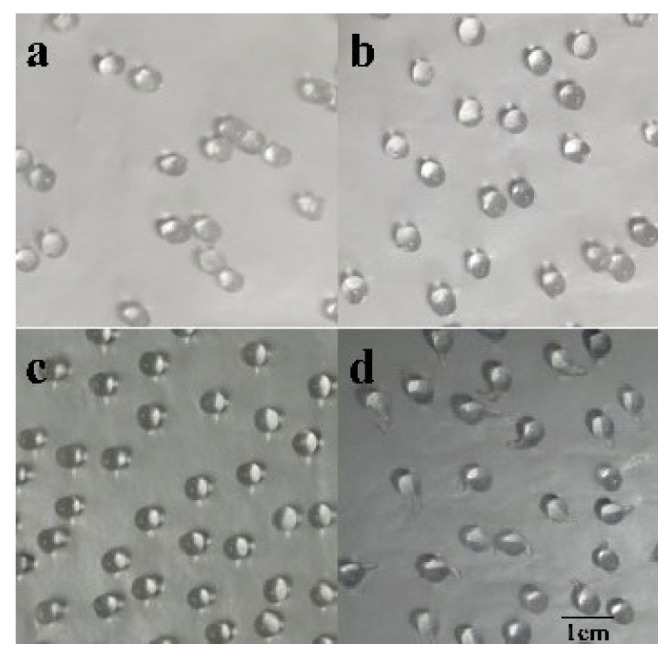
The immobilized enzyme morphology as a function of sodium alginate %-age. (**a**): 1.0% (**b**): 2.0% (**c**): 3.0% (**d**): 4.0%.

The morphology of sodium alginate beads with different concentrations of sodium alginate is shown in Figure 8. It was observed that the particle size of 3% sodium alginate beads with low hardness is more uniform, and the beads are easy to drop and their roundness is appropriate. There is no obvious tailing on the surface of the 3% sodium alginate beads, which has good viscoelasticity, good transparency and excellent mechanical strength. However, the particle size of other immobilized enzymes prepared by using different concentrations of sodium alginate is not uniform with low roundness.

**Figure 9:**
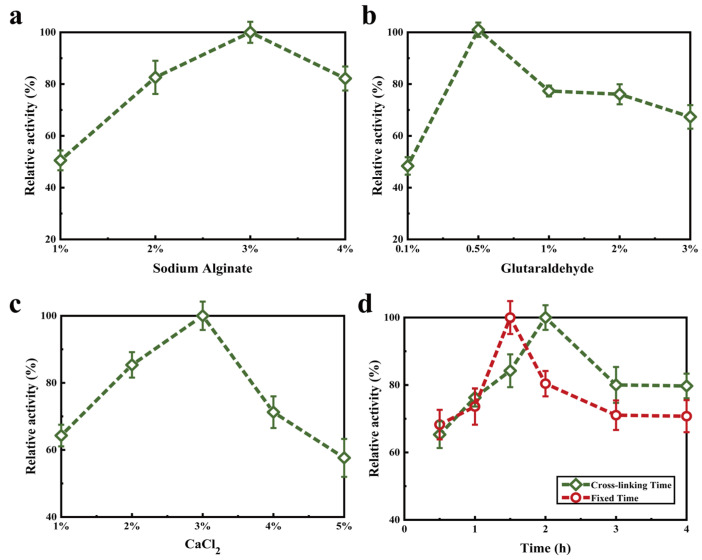
Effects of sodium alginate concentration (**a**), glutaraldehyde concentration (**b**), CaCl_2_ concentration (**c**), fixing and cross-linking time (**d**) on κ-selenocarrageenase activity in immobilization process.

Too high a concentration of sodium alginate would limit the diffusion of both substrate and product. Instead, too low a concentration of sodium alginate would lead to the loss of enzyme molecules from the gel space. As shown in Figure 9a, the relative enzyme activity increased with the increase in the concentration of sodium alginate up to 3.0%. When the concentration was >3.0%, however, the enzyme activity decreased. Because the gel formed between sodium alginate and free calcium ions, the enzyme was limited to react with κ-selenocarrageenan. The crosslinking process can restore damaged reticular structure, avoid enzyme shedding and improve its reusability [33]. Glutaraldehyde is a protein denaturing agent and also a crosslinking agent. In the range of 0.1–0.5% glutaraldehyde, the activity of immobilized enzyme increased continuously (Figure 9b). Excessive glutaraldehyde concentration can lead to excessive crosslinking and inactivation of the enzyme, leading to activity decrease of >0.5%. Alginate can combine with calcium ions to form a special EGG-box structure to immobilize the enzyme [34]. When the concentration of calcium ions is too low, the embedding effect is poor. In contrast, excessive calcium ions covering the gel surface will hinder the interaction between enzyme and substrate. Therefore, the enzyme activity reached the highest when the concentration of CaCl_2_ reached 3% (Figure 9c). Too long fixed time may aggravate the action of calcium ions on the enzyme, hinder the reaction of substrate and lead to the decrease in enzyme activity. If the fixation time is too short, the gap of the gel is enlarged, and the enzyme is not firmly bound to the substrate [35]. As can be seen from Figure 9d, the maximum activity for the fixed time scenario is 1½ h and the cross-linking time at 2 h. After optimization, the activity recovery rate of immobilized enzyme, i.e., the residual enzyme activity, was 62.83%.

**Figure 10:**
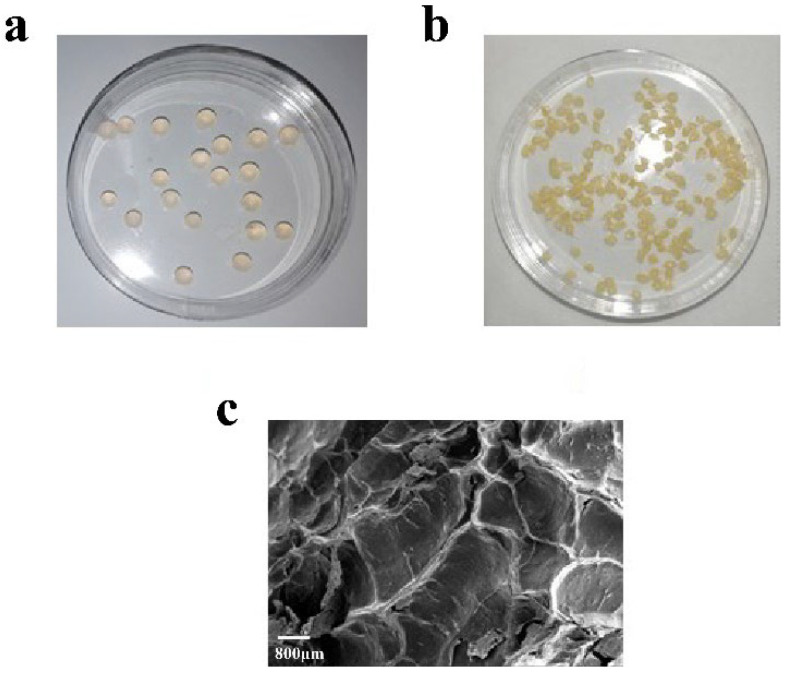
The immobilized enzyme particles morphology. (**a**) Immobilized enzyme particles; (**b**) lyophilized immobilized enzyme particles; (**c**) the lyophilized particle under scanning electron microscopy (SEM).

Figure 10 shows the morphology of alginate sodium-immobilized enzyme. The milky white immobilized enzyme has uniform particle size and smooth surface. The freeze-dried alginate sodium-immobilized enzyme had loose network structure (Figure 10c).

### 2.6. Stability of Immobilized κ-Selenocarrageenase

**Figure 11:**
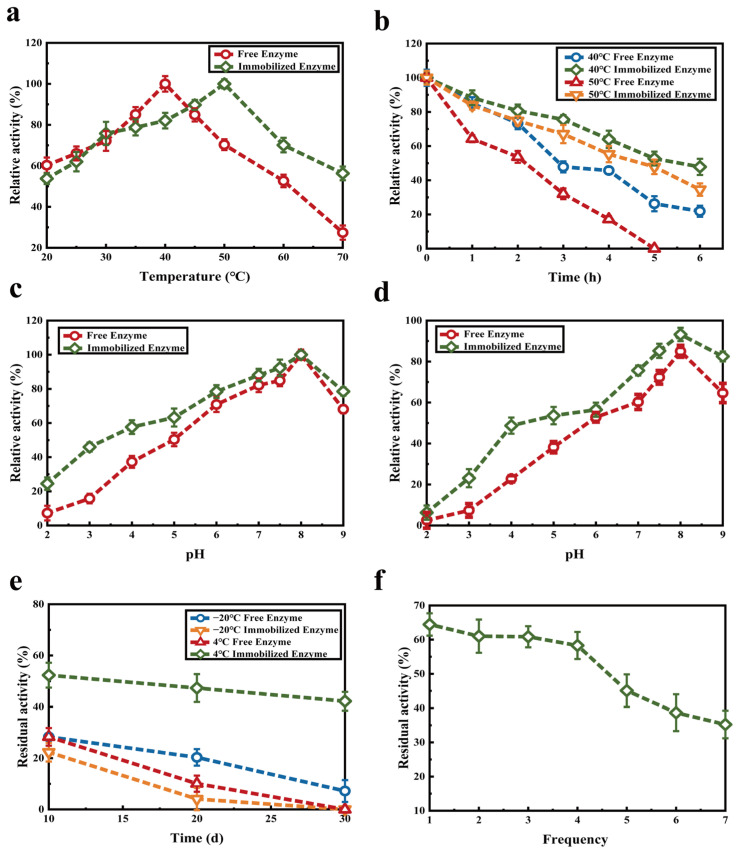
Storage stability test of immobilized enzyme. (**a**) Effect of temperature on the activity of immobilized enzyme; (**b**) thermal stability of immobilized and free enzymes; (**c**) effect of pH on immobilized enzyme activity; (**d**) pH stability of immobilized and free enzymes; (**e**) storage stability of immobilized and free enzymes at −20 °C and 4 °C; (**f**) operational stability of immobilized enzyme.

As shown in Figure 11a, the immobilized κ-selenocarrageenase exhibited higher thermal stability with optimum temperature of 50 °C which is 10 °C higher than that of free enzyme. Considering the poor thermal stability of this free enzyme, immobilization is of great significance for its application. When κ-selenocarrageenase molecules were immobilized by sodium alginate, the carrier would produce steric and shielding effects. The movement of the enzyme molecules is blocked by the alginate gel network, which greatly increases the ability of the enzyme to resist the external environment [21,36]. The immobilized κ-selenocarrageenase has higher stability at elevated temperatures than the free enzyme as reported (Figure 11b). The optimum pH of both free and immobilized enzyme was 8.0 (Figure 11c). When the free and immobilized enzyme were placed in the buffer solution of pH 2.0–9.0 for 3.0 h, respectively, the free enzyme was more sensitive. The activity of immobilized enzyme preparation remained >80% after being placed at 4 °C, pH 7.5–9.0 for 3.0 h, showing good tolerance to alkaline environment (Figure 11d).

As shown in Figure 11e, the rate of loss of immobilized and free enzymes increased with the extension of storage time at −20 °C. After 20 days of storage, the residual activity of the free enzyme was 20.31%; however, the activity of the immobilized enzyme was almost completely lost. The reason may be that the water in the immobilized enzyme is frozen at −20 °C, forming many ice grains. On the one hand, the ice grains can reduce the stability of enzymes and thus affect the enzyme activity. On the other hand, ice particles change the internal structure of sodium alginate particles, affecting the internal diffusion and thus the overall speed of the catalytic reaction. Instead, at 4 °C, the activity rate of loss of immobilized enzyme was significantly lower than that of free enzyme. This may be because the spatial configuration of the enzymes embedded in sodium alginate is more stable and the mechanical strength is enhanced [37]. After 30 days, the residual enzyme activity of the immobilized enzyme remained >40%, while the free enzyme was inactivated, indicating that the storage stability of the immobilized enzyme was better than free enzyme at 4 °C. The immobilized enzyme benefits the repeated, continuous, or batch uses of enzymes, and as a result reduces production cost. The reusability of the immobilized enzyme is an indispensable parameter for industrial applications. Figure 11f shows the activity of immobilized enzyme after repeated use. After being recycled four times, the immobilized enzyme activity decreased to 58.28%. It showed better reusability than that of κ-carrageenase immobilized onto magnetic iron oxide nanoparticles, the enzyme activity of which was only 43.5% after four cycles [38]. The reason for the enzyme activity loss may be that the immobilized enzyme granules are broken, which leads to the leakage of the enzyme, or some free enzyme which is not firmly bound to the carrier leaks. The experimental results showed that the alginate immobilized enzyme was feasible and could be used many times.

### 2.7. Selenium Content of the Enzymatic Degradation Product by κ-Selenocarrageenase Preparations

As shown in Table 4, the average selenium content of κ-selenocarrageenan was 55.73 µg/g. The organic selenium content of the degradation products by κ-selenocarrageenase in either lyophilized or immobilized form were 43.45 µg/g and 47.06 µg/g, respectively. The results showed the selenium content of κ-selenocarrageenan oligosaccharides degraded by the immobilized enzyme was 8.3% higher than that of degraded by the lyophilized enzyme. The immobilized enzyme catalyzes a heterogeneous reaction while the lyophilized enzyme degrades carrageenan in homogeneous reaction [39]. For the heterogeneous reaction catalyzed by an immobilized enzyme or other porous catalysts, the whole reaction process consists of five reaction steps as follows [39,40,41]: (a) the substrate in bulk fluid of reaction medium reaches on the outer surface of the catalyst particles through external diffusion; (b) the substrate on the outer surface enters the inner surface of the particles through internal diffusion; (c) the substrate on the inner surface makes contact with the catalyst site or the enzyme immobilized on the inner surface for a catalytic reaction; (d) the product generated comes to the outer surface through internal diffusion; (e) the product on the outer surface reaches into bulk fluid of reaction medium through external diffusion. It is speculated that the high viscosity of liquid due to the high concentration of κ-carrageenan increases the external diffusion resistance and reduces the external diffusion rate. Internal diffusion refers to the movement of molecules in the tiny pores of the catalyst. Substrate molecules diffuse from the immobilized enzyme particle surface into the particle interior, and come in contact with enzyme molecules [42]. Small carrageenan molecular weights lessen the internal diffusion resistance; instead, the internal diffusion resistance of large molecular weights is large [41]. Immobilized enzymes react mainly with small molecular weight carrageenan, which is different from lyophilized enzymes. The low molecular weight carrageenan may contain higher selenium content, so catalytic degradation products by the immobilized enzyme have higher selenium content.

## 3. Materials and Methods

### 3.1. Chemicals and Reagents

Glucose, ammonium sulfate, polyethylene glycol, mannitol, D-Trehalose anhydrous, galactose, glycine, 3,5-dinitrosalicylic acid, seignette salt and phenol were purchased from Sinopharm Chemical Reagent Co., Ltd. (Shanghai, China). κ-selenocarrageenan were purchased from Qingdao Pengyang Technology Development Co., Ltd. (Qingdao, China).

### 3.2. Microbial Strain and Cultivation

*Pseudoalteromonas sp*. Xi13 was isolated from the floating ice in Antarctica. The strain was cultivated on the 2216E medium (5 g tryptone L^−1^, 1 g yeast extract L^−1^ and 0.1 g ferric phosphate L^−1^) and the κ-selenocarrageenan screening plate (5 g tryptone L^−1^, 1 g yeast extract L^−1^, 0.1 g ferric phosphate L^−1^, 15 g κ-selenocarrageenan L^−1^ and 15 g agarl L^−1^).

### 3.3. Preparation of κ-Selenocarrageenase and Its Activity Assay

The fermentation broth was centrifuged at 7500 rpm at 4 °C after incubation overnight at 37 °C and then centrifuged at 12,000 rpm at 4 °C for 15 min to obtain the supernatant containing extracellular enzymes. The cell sediment was resuspended with phosphate buffered saline (PBS) solution and broken by ultrasound for 20 min. The cell disruption solution was centrifuged at 12,000 rpm at 4 °C for 15 min to obtain the supernatant containing intracellular enzyme. The κ-selenocarrageenan screening plate was divided into four regions (a: intracellular enzyme of Xi13; b: extracellular enzyme of Xi13; c: bovine serum protein; d: PBS), and then placed at 25 °C for 12 h to investigate κ-selenocarrageenan degradation effect.

Using κ-selenocarrageenan as substrate, the activity of κ-selenocarrageenase was measured by DNS (Dinitrosalicylic acid) method, and the absorbance was measured at 500 nm [43]. One unit of enzyme activity was defined as the amount of enzyme required to produce 1 mg oligosaccharides per minute at pH 7 and 25 °C. The activity of immobilized enzyme preparation was measured by reaction with 0.2% κ-selenocarrageenan substrate.

### 3.4. Optimization of Fermentation Conditions of Strain Xi13 by RSM

Three factors were selected: κ-selenocarrageenan concentration (**A**), Ca^2+^ concentration (**B**) and temperature (**C**), according to the central combination test design principle of RSM [19] and Wang’s research [17]. The activity value of κ-selenocarrageenase was selected as the response value *Y*. We used 3 factors and 3 levels (Table 5). The experimental design and results are shown in Table 6.

### 3.5. The Enzymatic Properties of κ-Selenocarrageenase

With the inactivated enzyme as control, the activity of κ-selenocarrageenase was measured in 0.2% κ-selenocarrageenan solution, at 20, 25, 30, 35, 40, 45, 50, 60, and 70 °C, respectively. To determine the optimal pH of κ-selenocarrageenase, the enzyme solution was mixed with PBS solution with different pH values in equal proportion and placed at 4 °C for 3.0 h to measure the relative enzyme activity. Ba^+^, Na^+^, Ca^2+^, Cu^2+^, Zn^2+^, K^+^, Mg^2+^, and Mn^2+^ were added into κ-selenocarrageenase solution to a final concentration of 5.0 mmol/L. The relative enzyme activity was calculated with the unit of enzyme activity of 100% when no metal ions were added. Protein inhibitors such as EDTA (Ethylene Diamine Tetraacetic Acid), β-mercaptoethanol, DTT (dithiothreitol) and PMSF (Phenylmethylsulfonyl fluoride), denaturant (SDS, sodium dodecyl sulfate), and surfactant (Tween-80) were added to the enzyme solution, and the final concentration was 1.0 mmol/L. The relative enzyme activity, without adding any additive, was referenced as 100% per unit of enzyme activity.

### 3.6. Preparation of Lyophilized κ-Selenocarrageenase

The obtained crude enzyme solution was pre-frozen until the tempreture of it reached −20 °C, −80 °C, respectively. At the same time, the same volume of crude enzyme solution was directly pre-frozen with liquid nitrogen. Then, the above samples were freeze-dried under vacuum for 24 h. The crude enzyme solution was pre-frozen at −80 °C for 6 h, 12 h, 18 h, and 24 h, respectively, and then freeze-dried under vacuum for 24 h to investigate the effect of pre-freezing time on the κ-selenocarrageenase activity. To investigate the effect of thickness of the enzyme solution on κ-selenocarrageenase activity, the crude enzyme solution was pre-frozen at −80 °C for 12 h, and the samples were freeze-dried at a thickness of 3.0, 4.0, 5.0, 6.0, 7.0 mm, respectively. The drying time also affected the κ-selenocarrageenase activity. With the thickness of 4.0, 5.0, and 6.0 mm, respectively, the samples were pre-frozen at −80 °C for 12 h and then freeze-dried for 0, 5, 10, 15, 20, 25, and 30 h, respectively. The activities of enzymic preparations under the above conditions were measured.

To investigate the effects of temperature, air and light on the stability of the enzyme, the enzyme preparations were stored at −20 °C or 4 °C under different conditions: exposed to or isolated from the air and light, or a combination of the two, respectively. The enzyme activities of free enzyme and lyophilized enzyme were measured at 40 °C for a period of time and at different pH, respectively. The enzyme activity of 0.5 g vacuum-packed lyophilized enzyme was measured every 10 days at −20 °C, 4 °C, 25 °C and 37 °C. Generally, food additives need to meet the requirements of asepsis while ultraviolet irradiation (UV) is usually used for sterilization; thus, the preparations were irradiated under UV with 100 μm/cm^2^ at 10, 20, 30, 40, 50, 70, and 90 min, respectively; then, the enzyme activity was measured.

### 3.7. Preparation of Immobilized κ-Selenocarrageenase

We mixed 3.0% sodium alginate solution and enzyme solution in equal proportion; then, 2.0% glutaraldehyde solution was added to the mixed solution, stirred thoroughly, and incubated for 1.0 h for cross-linking. The above solution was injected into 2.0% CaCl_2_ solution to form smooth pellets. After standing and hardening for 1.0 h, the beads were washed three times with PBS solution. The immobilized enzyme was obtained by removing surface moisture of the beads, then stored at 4 °C.

The free enzyme and immobilized enzyme were kept in buffer with different pH at 40 °C for 6.0 h and for 3.0 h, respectively. The free and immobilized enzyme were stored at pH 8.0 at −20 °C and 4 °C for 30 days, respectively. The activities of free enzyme and immobilized enzyme were measured at different time intervals.

### 3.8. Determination of the Selenium Content in Selenium Oligosaccharides

The reaction solution after degradation by the two enzymes was dialyzed against water dialysis bag (cut-off Mw: 3500 Da), concentrated by rotary evaporation, and dried. Dried selenium oligosaccharides in the amount of 1.0 g from all selenite treatment groups were digested with 15 mL of a mixture of HNO_3_, HClO_4_ andHSO_4_ (*v*/*v*/*v*, 4:1:1) at 100 °C for 24 h in a digestion tube until the solution became clear. The digested sample was cooled and diluted to 20 mL with deionized water. The final product was analyzed at 335 nm using UV spectrophotometer (721-100, TIANPU Co., Ltd., Beijing, China) to determine the selenium content. Selenium standard solution (10 µg/mL) was added to 2 mL of 1% o-phenylenediamine hydrochloride solution and diluted to 40 mL with water, respectively. The solution was adjusted to pH 2.0, reacted in the dark for 1.0 h, and extracted with toluene. Standard selenium solutions with different concentrations (20, 40, 60, 80, 100 µg/mL) were used to develop a standard curve. Inorganic selenium and organic selenium were separated by ethanol precipitation. After the selenium oligosaccharide was dissolved, the content of inorganic selenium in the supernatant was determined. The organic selenium was calculated by subtracting inorganic selenium content from total selenium content.

## 4. Conclusions

In the present study, the fermentation conditions for producing κ-selenocarrageenase from *Pseudoalteromonas sp*. Xi13 was optimized using RSM. The maximum κ-selenocarrageenase activity was increased by 1.4 times, reaching 8.416 U/mL under the optimum conditions: 1.6% κ-selenocarrageenan, 3.7 mmol/L Ca^2+^, and 33 °C. With trehalose as a lyophilized protective agent, the enzyme was freeze-dried under the optimized conditions which are as follows: −80 °C pre-freeze temperature, 12 h pre-freeze time, 2% trehalose, 5 mm thickness of enzyme solution layer. With the increase in drying time, the activity recovery rate of the lyophilized enzyme decreased gradually and tended to be stable at 25 h, which was finally >70%. After 30 days of storage, activity recovery rate of lyophilized enzyme was 36.22%. The selenium content of κ-selenocarrageenan oligosaccharides degraded by the lyophilized enzyme was 43.45 µg/g. With glutaraldehyde as cross-linking agent, the enzyme was immobilized using sodium alginate as carrier under immobilization conditions which are as follows: 3.0% sodium alginate, 0.5% glutaraldehyde, 3.0% CaCl_2_, 90 min cross-linking time and 120 min fixation time. The activity recovery rate of the immobilized enzyme was 62.83%. The thermal stability and pH tolerance of the immobilized enzyme were significantly better than those of the free enzyme. The enzyme activity remained at 58.28% after reuse four times. The selenium content of κ-selenocarrageenan oligosaccharides degraded by the immobilized enzyme was 47.06 µg/g, 8.3% higher than that degraded by the lyophilized enzyme. All of this work will be useful as a foundation for the industrial production of selenium oligosaccharides.

## Figures and Tables

**Table 1:** Analysis of variance of regression equation.

Factor	QS	DOF	MS	*F*-Value	*Pr* > *F*
Model	31.14	9	3.46	28.04	0.0001 ***
A	1.51	1	1.51	12.22	0.0101 *
B	1.23	1	1.23	9.99	0.0159 *
C	1.15	1	1.15	9.35	0.0184 *
AB	0.85	1	0.85	6.92	0.0339 *
AC	0.83	1	0.83	6.73	0.0357 *
BC	0.13	1	0.13	1.06	0.3371
A^2^	16.09	1	16.09	130.36	<0.0001 ***
B^2^	6.57	1	6.57	53.26	0.0002 ***
C^2^	0.82	1	0.82	6.64	0.0366 *
Residual	0.86	7	0.12		
Lack of Fit	0.6	3	0.2	3.04	0.1552
Pure Error	0.26	4	0.066		
Cor Total	32.01	16			

* Significant at *p* < 0.05; *** Significant at *p* < 0.001.

**Table 2:** Verification test.

	Experimental Enzyme Activity (U/mL)	Mean Value (U/mL)	Predicted Enzyme Activity (U/mL)
1	8.372	8.416	8.495
2	8.407		
3	8.468		

**Table 3:** Relation between liquid thickness and Dehydration time.

Enzyme Solution Thickness (mm)	Dehydration Time (h)	Enzyme Solution/Dehydration Time (mL/h)	Relative Enzyme Activity (%)
3	15	10.69	83.63
4	20	10.79	93.24
5	24	11.3	100
6	30	10.99	83.65
7	35	10.97	78.62

**Table 4:** Determination of the selenium content.

	A (µg/g)	B (µg/g)	C (µg/g)
	56.25	43.62	47.3
Experimental Value	55.64	43.07	47.01
	55.3	43.68	46.86
Mean Value	55.73	43.45	47.06

A: κ-selenocarrageenan; B: lyophilized enzyme preparation degradation products; C: immobilized enzymes degradation products.

**Table 5:** Factors and levels of response surface analysis.

Level	Factor		
	A: κ-Selenocarrageenan %	B: CaCl_2_ mmol/L	C: Temperature °C
−1	1	2	30
0	1.5	4	35
1	2	6	40

**Table 6:** Experiment design and result of response surface analysis.

Run	A	B	C	Y (U/mL)
1	1.5	6	30	6.442
2	1	2	35	4.395
3	1.5	4	35	8.731
4	1.5	2	30	7.163
5	2	4	30	7.342
6	1.5	4	35	8.301
7	1	6	35	4.832
8	2	6	35	4.932
9	1.5	2	40	7.198
10	1.5	4	35	8.301
11	1.5	4	35	8.301
12	2	4	40	5.238
13	1	4	30	5.718
14	2	2	35	6.343
15	1	4	40	5.437
16	1.5	6	40	5.753
17	1.5	4	35	8.014

## Data Availability

The data presented in this study are available in article.

## Sample Availability

Samples of the compounds are available from the authors.
